# Celecoxib does not appear to affect prosthesis fixation in total knee replacement: A randomized study using radiostereometry in 50 patients

**DOI:** 10.1080/17453670902804976

**Published:** 2009-02-01

**Authors:** Andreas Meunier, Per Aspenberg, Lars Good

**Affiliations:** Division of Orthopedics, Department of Clinical and Experimental Medicine, Faculty of Health Sciences, Linköping UniversitySweden

## Abstract

**Background and purpose** After joint replacement, a repair process starts at the interface between bone and cement. If this process is disturbed, the prosthesis may never become rigidly fixed to the bone, leading to migration—and with time, loosening. Cox-2 inhibitors are widely used as postoperative analgesics, and have adverse effects on bone healing. This could tamper prosthesis fixation. We investigated whether celecoxib, a selective Cox-2 inhibitor, increases prosthesis migration in total knee replacement (TKR).

**Methods** 50 patients were randomized to either placebo or celecoxib treatment, 200 mg twice daily, for 3 weeks after TKR (NexGen; Zimmer). Maximum total point motion (MTPM) of the tibial component was measured after 2 years using radiostereometric analysis (RSA). In addition, range of motion, pain, and, subjective outcome were evaluated.

**Results** No differences in prosthesis migration, pain scores, range of motion, and subjective outcome were found after 2 years. Confidence intervals were narrow.

**Interpretation** It is unlikely that Celecoxib increases the risk of loosening, and it may be used safely in conjunction with TKR.

## Introduction

Cyclooxygenase-2 (Cox-2) is involved in the bone healing process and is inhibited by both selective and non-selective inhibitors. There is strong evidence from animal studies that Cox-2 inhibitors delay bone healing in diaphyseal fracture models (Zhang et al. 2002, Seidenberg and An 2004, Gerstenfeld et al. 2007, Saudan et al. 2007), and small effects have also been found in a stable fixation model in metaphyseal bone in rats (Meunier and Aspenberg 2006). In humans, there is strong evidence that Cox inhibitors inhibit heterotopic bone formation (Wahlstrom et al. 1991, Saudan, et al. 2007) and they also appear to delay bone healing in diaphyseal fractures (Giannoudis et al. 2000, Burd et al. 2003) and spinal fusion (Reuben et al. 2005). Even so, Cox-2 inhibitors are being increasingly used in pain management after orthopedic surgery (Reuben et al. 2002).

Both after cemented and uncemented joint replacement, a bone repair process starts at the bone interface because of the inevitable bone damage (Larsen and Ryd 1989). The extent to which this process is influenced by Cox inhibitors is unclear. If healing is disturbed, the prosthesis may never become rigidly fixed to the bone, leading to migration—and with time, loosening. An elevated number of revisions, although with borderline statistical significance, were found following total hip replacement (THR) 10 years after receiving a Cox inhibitor as prophylaxis for heterotopic bone formation (Persson et al. 2005). Cox inhibitors are, however, effective analgesics and may reduce the inflammatory response to surgery; they have also been shown to increase the range of motion in the early phase of rehabilitation (Reuben et al. 2002). Thus, Cox inhibitors have gained wide popularity as postoperative analgesics. The possible risk of impaired TKR survival has never been investigated, which was the reason for this study.

Our hypothesis was that celecoxib, a selective Cox-2 inhibitor, increases prosthesis migration in total knee replacement. Migration was measured by radiostereometric analysis (RSA), which constituted the primary evaluation variable. Secondary variables were pain, range of motion, and subjective outcome.

## Patients and methods

The study was designed as a randomized, placebo-controlled, double-blind trial, and was performed in accordance with the ethical standards of the Helsinki Declaration of 1975, as revised in 2000. It was approved by the regional ethics committee (no. 03-286) and the Medical Product Agency in Sweden (no. 151:2003/47246). The study was conducted from March 2004 through February 2005 at the Department of Orthopedic Surgery, Linköping University Hospital, Sweden. 50 patients suffering from osteoarthritis, who met the inclusion criteria below, were consecutively recruited from the waiting list for elective primary unilateral TKR (Table 1). The inclusion criteria were: age 50–80 years, ASA I–II, and capacity to give informed consent. The exclusion criteria were: a history of coagulopathy or of a thromboembolic event, plasma creatinine > 100 mmol/L in women and > 115 mmol/L in men, acute infection, malignant disease, unstable angina, myocardial or cerebral infarction 1 year or less before operation, and allergy to NSAIDs or sulfonamides. All ongoing NSAID therapy was discontinued 7 days before surgery and for 3 weeks postoperatively.

**Table 1.** Patient characteristics

	Placebo (n = 25)	Celecoxib (n = 25)
Sex, M/F	14/11	8/17
Age, years (SD)	69 (8)	68 (6)
Time of surgery, min (SD)	87 (11)	80 (9)

Capsules containing either placebo or celecoxib (200 mg) were prepared by Apoteket AB (Stockholm, Sweden). Sets of capsules were randomly numbered 1–50 by a computer generator in 5 blocks of 10 sets. The content of every set of capsules was automatically documented by computer, printed out, and finally stored in a sealed envelope that was numbered according to the randomization. A research nurse administered the numbered sets of capsules consecutively to the patients and the number of each set was traced on the evaluation form for each patient. Thus, all 50 patients randomly received either placebo or celecoxib (200 mg) orally 1 h preoperatively, and then twice daily for 3 weeks.

All patients received NexGen prostheses (Zimmer), fixed to both the femur and the tibia with Palacos cum gentamicinum bone cement (Heraeus Medical, Germany). After partial exsanguination of the limb, a pneumatic tourniquet on the thigh was inflated to 300 mm Hg. The operation was performed by 1 of 3 surgeons who were well experienced in this procedure. 1 of them (AM) performed 42 of the 50 operations. Intraoperatively, the tibial component, an all-polyethylene construct, and the proximal tibia were marked with tantalum balls (diameter 0.8 and 1.0 mm) for later radiostereometric analysis. The joint was drained with a single closed suction drain for 24 h postoperatively. Rehabilitation was initiated at the ward by a physiotherapist and continued under supervision of physiotherapists for 3–8 weeks (depending on individual needs) after discharge from the hospital.

We recently reported the 1-year follow-up results of the clinical outcome variables (Meunier et al. 2007).

### RSA

Index RSA radiography was conducted on the second day after surgery, after weight bearing had been initiated, and served as a baseline for subsequent measurements of migration that were performed after 6, 12, and 24 months. The primary evaluation variable was maximum total point motion (MTPM). It describes the vector length of the one tantalum ball in the tibial component with the largest range of motion without taking the direction in account. In addition, we report rotation and translation of the rigid body formed by the tantalum balls in the tibial prosthesis in relation to the reference body in the skeleton of the proximal tibia. Rotation about the x-axis denotes forward or backward tilting of the prosthesis, rotation about the y-axis denotes inward or outward rotation, and rotation about the z-axis denotes varus-valgus tilting. Accordingly, translations along the x- and z-axes denote movement of the prosthesis in mediolateral and anteroposterior directions, respectively. Translation along the y-axis denotes sinking of the prosthesis. The position of the tantalum ball was measured and evaluated from digitized radiographs using RSA software (UmRSA; RSA Biomedical, Umeå, Sweden). If the mean error of the rigid body fitting exceeded 0.35 mm, the software excluded the tantalum ball that caused the lack of rigidity in the rigid body, because this particular ball was judged not to be fixed in the rigid body any more.

The conditioning number (CN) is a gauge of measurement quality of the rigid body formed by the tantalum balls. The lower the CN, the greater is the volume of the rigid body formed by the balls and the better the quality of measurement. If the CN exceeds 90, measurement becomes inexact and that particular radiograph is excluded from further evaluation.

Preoperatively and after 3, 12 and 24 months, pain was rated with a visual analog scale (VAS) where 0 represents no pain and 10 represents the worst possible pain. The range of knee motion (ROM) was recorded by 1 of 2 physiotherapists preoperatively and after 3, 12, and 24 months. Subjective outcome was assessed preoperatively and after 24 months using the knee injury and osteoarthritis outcome score (KOOS).

### Statistics

The primary endpoint, maximum total point motion (MTPM), has a minimal clinically relevant value of 0.2 mm, as suggested by Ryd et al. (1995). Hence, the study was powered to detect a difference in MTPM of 0.2 mm between the groups. With a = 5% and b = 20%, and the SD for MTPM from a comparable study (Hilding et al. 2000), a study size of 18 patients per group was calculated. To compensate for possible exclusion, we chose a study size of 25 patients per group.

MTPM at 24 months was analyzed using Student’s t-test. To avoid multiplicity-testing problems, the secondary outcome variables are only reported descriptively as graphs or arithmetic means (SD) and with the 95% confidence interval (CI) for group differences. In addition, for segment motion a Levene’s test was performed to detect significant differences in SD. Significance was assumed at p-values less than 0.05.

## Results

2 patients were excluded from follow-up: 1 patient in the placebo group sustained a gastric ulcer, necessitating discontinuation of the study medication, and 1 patient in the celecoxib group had moved abroad and was not available for follow-up.

**Figure 1.**
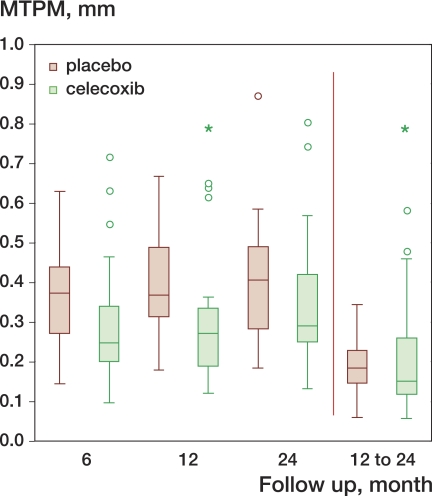
MTPM during follow-up, median (line) with interquartile range (boxes) and non-outliers range (whiskers), placebo, celecoxib, outliers (values, 1,5-3-fold larger than the interquartile range), * extremes (values > 3-fold larger than the interquartile range).

**Figure 2.**
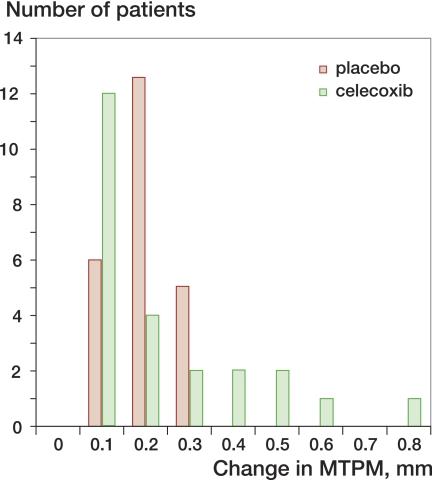
MTPM between 1 and 2 years.

The MTPM at 2 years was similar in both groups (celecoxib: 0.35 mm (0.17), placebo: 0.4 mm (0.15), CI for group difference –0.15 to 0.04) (Figure 1). The calculated confidence interval for the primary endpoint indicates that any real difference in outcome between the groups, with 95% confidence, is less than the pre-specified minimal clinically relevant value. No significant differences of rotation and translation values were found between the two groups (Table 2). Migration (MTPM) between 1 and 2 years was 0.23 mm (0.18) in the celecoxib group and 0.2 mm (0.07) in the placebo group (CI for group difference –0.5 to 0.1). There were a few more outliers in the celecoxib group (Figure 2). The conditioning number for the prosthesis segment was 21 (10) and for the reference segment 20 (5). The mean error of rigid body fitting for the prosthesis segment was 0.11 (0.05) and for the reference segment 0.17 (0.08).

**Table 2.** MTPM (mm) and segment motion (rotation in degrees and translation in mm) at 2 years

	Placebo		Celecoxib		95% CI		Levene’s test
	mean	SD	mean	SD	Min.	Max.	p-value
MTPM	0.40	0.15	0.35	0.17	–0.15	0.04	0.56
Rotation x (anterior–posterior tilt)	–0.24	0.24	–0.14	0.16	–0.02	0.22	0.10
Rotation y (inward–outward rotation)	–0.01	0.18	–0.01	0.17	–0.11	0.09	0.40
Rotation z (varus-valgus tilt)	0.01	0.15	0.00	0.12	–0.09	0.06	0.43
Translation x (medial–lateral translation)	0.00	0.05	0.00	0.06	–0.02	0.04	0.17
Translation y (subsidence – lift-off)	–0.01	0.12	0.01	0.13	–0.05	0.09	0.94
Translation z (anterior–posterior translation)	0.01	0.07	0.04	0.09	–0.01	0.08	0.27

**Figure 3.**
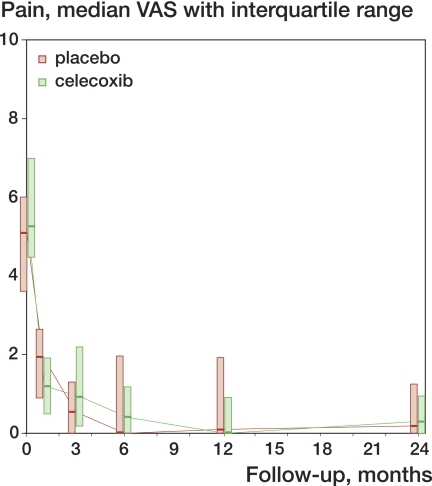
Pain during follow-up (median with interquartile range): placebo, celecoxib.

No differences were found in pain score, as measured with VAS. Due to skewed distribution, values had to be normalized by logarithmic transformation, which then showed similar means and standard deviations. In absolute values, the placebo group had a median value of 0.2 (interquartile range: 0–1.25) and the celecoxib group had a median value of 0.3 (interquartile range: 0–0.95) (Figure 3).

**Figure 4.**
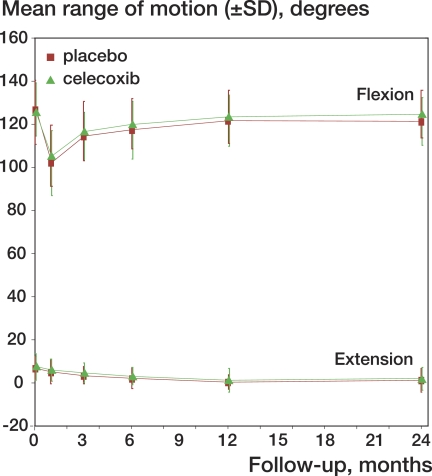
Development of range of motion during follow-up (mean ± SD): extension placebo, flexion placebo, extension celecoxib, flexion celecoxib.

Range of knee motion increased at the same rate in both groups, to the same total motion segment (flexion minus extension) (placebo: 121 degrees (SD 13); celecoxib: 123 degrees (SD 13); 95% CI: –10 to 6) (Figure 4).

**Figure 5.**
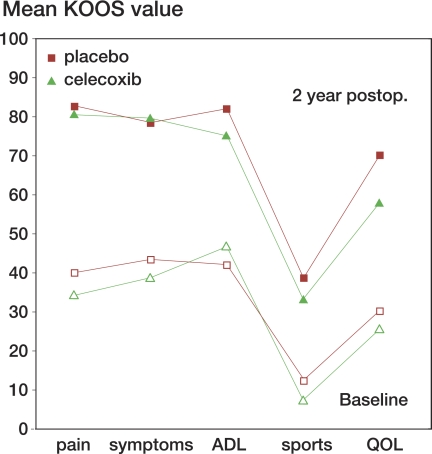
Mean KOOS values before and 2 years after TKR: placebo at baseline, celecoxib at baseline, placebo at 2 years, celecoxib at 2 years.

KOOS values increased to similar extents in both groups, from preoperatively to 2 years after surgery (Figure 5).

## Discussion

There were no differences in MTPM between the two groups, and no differences in the rigid body motion pattern. This suggests that celecoxib—and thus probably also other Cox-2 inhibitors—does not increase the risk of loosening after TKR. We noted a few more outliers with increased prosthesis motion in the celecoxib group, and these patients may be the ones with the highest risk of loosening (Figure 2). However, the study was powered to detect a difference in mean MTPM between the groups and not to detect a difference in the number of outliers. Moreover, the CI calculated is very narrow: less than the pre-specified minimal clinically relevant value of 0.2 mm. Cox inhibitors have been used for many years in pain management after TKR, but in spite of this, the frequency of revision surgery due to aseptic loosening has been shown to be quite low—about 5% after 10 years for all revisions—according to the Swedish knee arthroplasty register (Harrysson et al. 2004). The quality of our RSA examinations was good, with low conditioning numbers and low mean error of rigid body fitting. The magnitude of the MTPM is similar to that in comparable studies (Hilding et al. 2000, Norgren et al. 2004, Hyldahl et al. 2005, Hilding and Aspenberg 2006). Other factors that could contribute to motion, such as positioning of the prosthesis or osteoporosis, were not addressed in this study, but blinding and randomization should have eliminated the effects of such factors on the result.

We found no positive effects on range of motion, pain, and subjective outcome (as measured with the KOOS 2 years after TKR) when celecoxib had been given in the early rehabilitation phase. At 1 year, the results were similar (Meunier et al. 2007). Buvanendran et al. (2003) reported better ROM and less pain in patients treated with a Cox-2 inhibitor after TKR. In our study, we could not confirm those early positive effects after 1 or 2 years. If there was suppression of the inflammatory response to the surgical trauma in the early phase of rehabilitation, this did not lead to improved medium-term subjective and objective outcome.

In this study, we found that celecoxib given in the perioperative and postoperative phases of TKR did not lead to an increase in prosthesis migration 2 years after surgery. Celecoxib can safely be given as part of postoperative pain management, but there appears to be no long-term benefit regarding pain, range of motion, or subjective outcome.

AM participated in the study design process, performed all data collection and evaluation, and was the principal author of the manuscript. PA came up with the idea of the study, participated in study design, and helped in writing and revision of the manuscript. LG was responsible for the study design and all communication with the Medical Product Agency in Sweden. He supervised AM during the study, and participated in writing and revision of the manuscript.
